# Asymptomatic Bacteriuria in Pregnant Women in Outpatient Facilities

**DOI:** 10.5195/cajgh.2015.53

**Published:** 2015-02-19

**Authors:** Maral G. Nogayeva, Svetlana A. Tuleutayeva

**Affiliations:** Asfendiyarov Kazakh National Medical University, Almaty, Kazakhstan

**Keywords:** asymptomatic bacteriuria, pregnancy, antibiotic resistance, prevention

## Abstract

Urinary tract morbidity has increased by 7% in Kazakhstan between 2007 to 2011. Pregnant women with extragenital pathologies or kidney diseases had the greatest prevalence of morbidity. Asymptomatic bacteriuria (AB) is one of the most important risk factors of pyelonephritis development in pregnant women, and it can affect the course and outcome of pregnancy, delivery, and postnatal period. AB prevention requires prevention of pregnancy complications including early diagnostic of urinary tract infections, timely optimization of therapy at outpatient facilities, and dynamic follow-up.

Extragenital pathology in pregnant women remains one of the most urgent issues in health care. Previous research has indicated that urinary system infections are identified only in 15 to 30 % of all cases.^1^ One particular risk factor associated with an increase of urinary system infections and related complications is asymptomatic bacteriuria (AB). AB is an important risk factor in the development of pyelonephritis in pregnant women. AB means that a certain amount of bacteria is identified in properly collected urine samples taken from those who have no signs or symptoms of urinary system diseases in two consecutive tests taken within an interval of 1 to 2 weeks.^2^,^3^ Even when no clinically significant pyelonephritis is observed, AB may result in pre-term delivery, development of anemia in pregnant women, preeclampsia, low weight of newborn child, or fetal intrauterine retardation.^2^,^4^,^5^

Although there are numerous complications associated with AB, little attention is paid to AB and latent pyelonephritis in pregnant women. Delayed diagnostics and inadequate treatment of pyelonephritis and AB can result in chronic kidney disease, and over time, in chronic renal insufficiency.^6^,^7^ The aim of this study was to examine the diagnosis and treatment of AB in pregnant women in city polyclinic no. 5 (CP 5).

## Methods

Medical examination was performed on 140 pregnant women between 19 and 42 years of age. These women were patients of the prenatal clinic of CP 5 in the Almaly District, Almaty, Kazakhstan. Informed consent was obtained from all participants.

The following tests were performed for all pregnant women: general medical examination, full blood count, general urine test, urine test by Nechiporenko method, bacterioscopic and urine culture test, antibiotics sensitivity of urinary microflora (performed at INVIVO® Laboratory), biochemical blood assay for total protein, sugar, ALT, AST, cholesterol, creatinine, urea, blood coagulability, and ultrasound examination of kidneys using ALOKA 1400 unit. Urinary infection was treated to ensure both effective eradication of the causative agent of the specific diseases and safety of the fetus. AB was treated based on gestational period, causative agent, and antibiotic sensitivity. Safety of the treatment was evaluated according to side effects, allergic reactions, and individual intolerance. Fetal ultrasound was performed before and after treatment. Evaluation of the effectiveness of therapy was evaluated at the beginning of therapy, 10 days post-therapy, and 30 days post-therapy. Bacteriological test results were evaluated during each visit on the following criteria: recovery (sterile urine culture or bacteria in the urine at a concentration of less than 10^3^ CFU / ml); persistence of infection (detection of the same pathogen in the urine at 10^3^ CFU / ml or more); and reinfection (detection in the urine of a new species of bacteria at 10^3^ CFU / ml or more during any visit).

## Results

The average age of the 140 participants was 33.2 ± 2.53 years. All participants with AB were classified by gestation periods: first trimester – 74 women (52.8%), second trimester – 62 (44.3%), and third trimester – 4 (2.9%).

**Figure 1:**
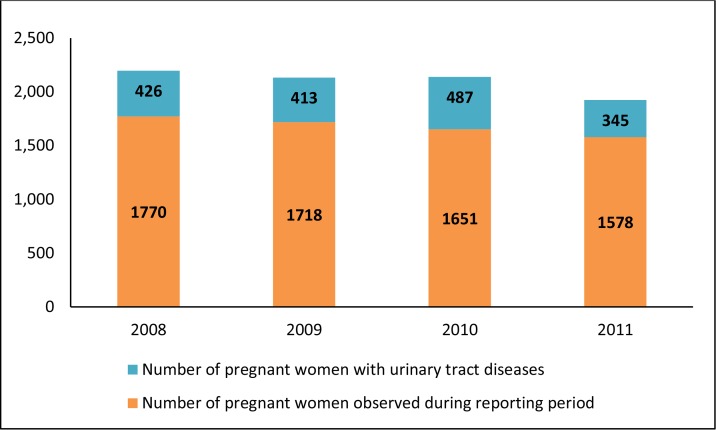
**Number of diagnosed urinary tract diseases among pregnant women from the Urinary System Diseases at CP5 prenatal clinic of Almaty from 2008 to 2011**

During the reporting period, there was an overall slight decrease in the number of diagnosed urinary tract diseases among pregnant women at CP 5; however, in 2010, the proportion of women with the disease went up to 29.5% compared to 24% in 2008–2009. These can be further seen in Figure 1.

Bacteriuria (≥105 cfu/ml) was detected in 38.5% of participants, and AB prevailed in the first trimester of pregnancy at a rate of 52.8%. Ultrasound examination of the kidneys identified a dilatation of the renal collecting system in 69 (49.3%) women and hydronephrotic transformation in 25 (17.9%) pregnant women with AB. 88% of participants with AB had asiderotic anemia of slight and medium degree. 2% had arterial hypertension, exhibited as general faintness, headaches, and dizziness, 27.8% had history of pyelonephritis, and 20.7% had previously diagnosed cystitis.

**Figure 2.**
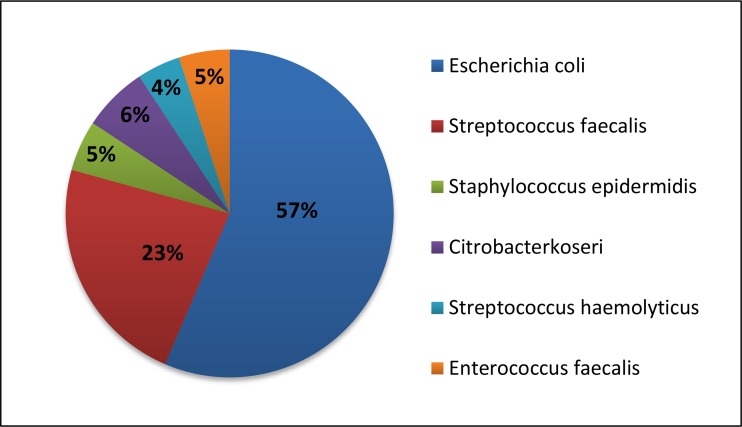
**Frequency of urinary bacterial microflora**

Tests for various types of urinary microflora and antibiotic sensitivity of the isolated microflora revealed optionally anaerobic commensal flora with prevalence of *Escherichia coli* and *Streptococcus faecalis*, which can be further explored in Figure 2*.* In all cases, the isolated microflora was resistant to more than one antibiotic, specifically Amoxiccillin; however, it was sensitive to Cephalosporins and Fluoroquinolones.

A complex therapy included antibacterial drugs (Cefuroxime, 750 mg, twice a day, 5 days), urinary antiseptics (Furazidin, 1 tablet, 4 times a day), vitamin and mineral supplements (depending on gestation period), and antiplatelet medication (Dipiridamol, 25 mg, 2 pills 4 times a day). Alternative therapy using third generation Cephalosporin was performed in 2 women against urinary pathology strains resistant to Cefuroxime. In women with AB, high resistance of microorganisms, and allergies, an alternative method of treatment was combined with herbal supplement Canefron N, (2 pills or 50 droplets, three times a day during a month).

As a result of the treatment, all pregnant women reported improvement of health, decrease of general weakness, and weight loss in 12 women (8.5%) for 14–17 days at 2–3 kg, due to the reduction or disappearance of edema. Recurrence of the treated bacteriuria and preeclampsia in our study was not observed.

## Discussion

According to annual reports of the Ministry of Health, overall urinary disease morbidity rate in population of Kazakhstan increased by 7 % from 2007 to 2011,^8^ which indicates the need for awareness as the majority of the cases are asymptomatic.

AB mainly prevails in the first trimester of pregnancy, which also confirmed the presence of chronic diseases in the urinary system before pregnancy. The main risk factors for the high prevalence of AB in the outpatient setting are cystitis and pyelonephritis in previous medical history,^1^,^6^ which should be also given special attention in family planning process. According to ultrasound examination of the kidneys, dilatation of the renal collecting system is a risk factor in renal inflammation, which means that the inflammatory process was prolonged. This can cause adverse events for the mother and fetus. *Escherichia coli* was the primary cause in most cases which are resistant to Amoxiclav. According to medical scientific sources, Citobacterkoseri is sensitive to such antibacterial drugs as Nifuroxazid and Levofloxacin, which are contraindicated for pregnant women. In our study, pregnant women took Cefalosporins, which are hardly active against this microorganism; however, they were combined with Canefron, which gave such positive outcome as elimination of causative agent.

Therapeutic tactics of treatment of pregnant women with AB includes timely complex treatment, control of sterility of urine at each visit to the doctor for the purpose of testing urine sterility in every trimester of pregnancy to prevent any complications in mothers and fetuses, and testing of the male partner for concealed bacteriospermia.
